# Changing Epidemiology of HIV in Mumbai: An Application of the Asian Epidemic Model

**DOI:** 10.5539/gjhs.v4n5p100

**Published:** 2012-08-05

**Authors:** Ram Manohar Mishra, Madhulika Dube, Damodar Sahu, Niranjan Saggurti, Arvind Pandey

**Affiliations:** 1 Population Council, New Delhi, India; 2 Maharshi Dayanand University, Rohtak, India; 3 National Institute of Medical Statistics, New Delhi, India

**Keywords:** Asian Epidemic Model, Human Immunodeficiency Virus, Mumbai

## Abstract

**Background:**

Mumbai is one of the most populous and high HIV prevalence cities in India. It has witnessed substantial changes in HIV-risk behaviors and a decline in HIV prevalence among high-risk groups during the past decade.

**Aim:**

To examine the changing pattern in the number of new HIV infections by transmission routes in Mumbai during 2000-2017.

**Methods:**

We used the Asian Epidemic Model by dividing the adult population (aged 15 and above) into seven subgroups: brothel-based and non-brothel based female sex workers (FSWs), heterosexual clients of FSWs, men who have sex with men/transgendered people (MSM), injecting drug users (IDUs), general women and general men. The MSM subgroup included homosexual and bisexual men.

**Results:**

New HIV infections among adults reduced by 86% during 2000-2010. The highest decline was among FSWs and their heterosexual clients (95%-98%), followed by MSM (82%), general women (77%), IDUs (51%) and general men (42%). Most new HIV infections during 2011-2017 are expected to occur among general women (1666) and general men (977) followed by MSM (715). Bisexual men were estimated to contribute about 14% of the new HIV infections among general women in 2010 and this proportion was estimated to increase to 19% in 2017.

**Discussion:**

HIV prevention programs for MSM and the general population need to be strengthened. Ensuring early detection of HIV, and higher levels of consistent condom use by HIV-infected men and women are essential to prevent new HIV infections in future.

## 1. Introduction

Mumbai is the capital of Maharashtra, which is one of the six high HIV prevalence states in India (National AIDS Control Organization, 2010). Mumbai is one of the most populous cities in the country, housing over 12 million people in 2011 (Registrar General of India, 2011). Since the inception of the HIV epidemic in the country, Mumbai has received extensive attention from epidemiologists, public health experts, social scientists and policy makers due to high HIV prevalence among both high-risk groups including female sex workers (FSWs), injecting drug users (IDUs), and men who have sex with men/ transgendered people (MSM) and low-risk women attending antenatal clinics (hereafter referred to as ANC women) (India Health Action Trust, 2010; Jain, John, & Keusch, 1994). Being the financial hub of the country, Mumbai has been a preferred destination for employment for thousands of young men from low HIV prevalence states (Jain et al., 1994; Saggurti, Verma, Jain, Achyut, & Ramarao, 2008; Singh, 2006). Many of these migrant men stay alone in the city away from their family, and a substantial proportion visit FSWs (Saggurti, Schensul, & Verma, 2009; Saggurti et al., 2008; Verma, Saggurti, Singh, & Swain, 2010). Research suggests that men with a non-resident wife practice high HIV risk behaviors in Mumbai and hence constitute an important bridge population to transfer the epidemic from high HIV prevalence areas to low HIV prevalence areas of the country (Saggurti et al., 2009; Verma et al., 2010). Mumbai also has major halt points where a large number of truckers wait for their consignments for a considerable period of time. The high-risk sexual behaviors of truckers in Mumbai has also been documented (Churi & Anjenaya, 2010; Zahiruddina, Gaidhanea, Shanbhagb, & Zodpeyb, 2011). Further, more than half of Mumbai’s population lives in slums (Singh, 2006) where men’s high-risk behavior has been reported (Saggurti et al., 2009; Schensul et al., 2006).

As in many places in India, the HIV epidemic in Mumbai is also believed to be largely driven by unprotected sex with FSWs; however, the presence of several high-risk groups in Mumbai has resulted in a more complex local HIV epidemic than elsewhere in the country (Avert Society, 2004, 2009; Family Health International, 2001; India Health Action Trust, 2010). This was reflected in the national response to the epidemic by setting up an autonomous society for implementing HIV prevention programs in Mumbai while in other parts of the country such programs are implemented by corresponding State AIDS Control Societies (National AIDS Control Organization, 2006a). Intensive HIV prevention interventions in Mumbai are being implemented by both government and non-government organizations. The intervention strategies include peer-led outreach for safe-sex counseling, distribution of condoms (free as well as socially marketed), treatment of sexually transmitted diseases (STD), needle and syringe exchange, community empowerment, and building a health enabling environment (Bill & Melinda Gates Foundation, 2008a; National AIDS Control Organization, 2006a; The Humsafar Trust, 2012). In Mumbai HIV prevention programs have been condom-centric and have focused primarily on FSWs, MSM, and truckers as most of the infections in India, apart from the north-eastern states, occur through the sexual route (Bill & Melinda Gates Foundation, 2008a, 2008b; National AIDS Control Organization, 2006a). The government provides treatment and care services to those infected with HIV, which are equally accessible to all individuals (National AIDS Control Organization, 2006a, 2010).

The major data sources to monitor the HIV epidemic in Mumbai are the annual HIV sentinel surveillance, facility-based data {National program for the prevention of parent-to-child transmission, National program for integrated counseling and testing centers, National program for antiretroviral therapy (ART)}, behavioral surveys, integrated behavioral and biological surveys, and size estimation and mapping data (India Health Action Trust, 2010; Sgaier et al., 2012). These data sources provide information on trends in the number of people in high-risk groups, HIV risk behaviors among high-risk and low-risk groups, and STD/ HIV prevalence among selected subgroups including FSWs, MSM, IDUs, and ANC women, and the number of people living with HIV who are receiving ART. Insights from these data sources suggest that during the past decade Mumbai has witnessed substantial changes in behavioral, social, cultural, and structural factors, which may affect the epidemiology of the HIV epidemic in the city. These changes include increase in consistent condom use by FSWs with clients from 62% in 2003 (Avert Society, 2004) to over 90% in 2009 (Avert Society, 2009; Indian Council of Medical Research & Family Health International, 2011), increase in consistent condom use by MSM in anal sex with men/ transgendered people from about 56% in 2001 (National AIDS Control Organization, 2006c) to about 80% in 2009 (Avert Society, 2009), decrease in the proportion of brothel-based FSWs from about 48% in 2001 (Family Health International, 2001) to about 30% in 2008 (India Health Action Trust, 2010), sharp decline in HIV prevalence among FSWs from over 50% in 2002 to less than 10% in 2008 (India Health Action Trust, 2010) and the introduction ART in 2004 (India Health Action Trust, 2010). The program monitoring data suggest that till April 2009, about 19500 people had ever started ART and 14604 were continuing on ART (India Health Action Trust, 2010).

Although the available data sources provide information on trends in HIV prevalence among FSWs, MSM, IDUs, and ANC women, they do not provide estimates of the number of new HIV infections in various subgroups, which is much more sensitive to the changing dynamics of disease transmission and a more accurate measure to detect program impact. Further, the distribution of new HIV infections by routes of transmission among various subgroups cannot be estimated and projected directly from the available information. This information, if available, could be of great value to policy makers in designing effective HIV prevention interventions and for the allocation of resources.

Mathematical models are often used to overcome the limitations of existing data sources by extracting such information from them. This paper uses the Asian Epidemic Model (AEM) to assess the changing epidemiology of HIV in Mumbai during 2000 to 2017 by: (1) estimating and projecting the number of new HIV infections among subgroups including brothel-based and non-brothel-based FSWs and their clients, MSM, IDUs, low-risk men and low-risk women, and (2) estimating the trend in distribution of new HIV infections among various subgroups by routes of transmission.

## 2. Methods

### 2.1 The Model

The AEM was developed by the East-West Center with support from USAID, UNAIDS, Family Health International, World Health Organization, and World Bank (Brown & Peerapatanapokin, 2004). The model considers HIV transmission among men and women aged 15 years or older (hereafter referred to as adults). People enter the population at age 15 and depart as a result of either AIDS-related or non-AIDS related death. The model allows the entire population to be divided into several subgroups according to their relevance to the socio-cultural setting and the nature of the local epidemic. It then mathematically replicates key processes driving HIV transmission among the defined subgroups.

We divided the adult women’s population into following three subgroups: (1) brothel-based FSWs (defined as FSWs who usually solicit from brothels); (2) non-brothel based FSWs (defined as FSWs who usually solicit from places other than brothels, such as streets, parks, lodges, home, and hotels); and (3) general women (defined as women who are not FSWs). FSWs were subdivided in two groups mainly because the sex work industry in Mumbai has changed substantially during the past decade. The adult men’s population was divided into the following four subgroups: (1) heterosexual clients (defined as heterosexual men who visit FSWs); (2) IDUs (defined as men who inject drugs); (3) MSM (defined as men who have sex with other men/ transgendered people); and (4) general men (defined as low-risk men who are not heterosexual clients, IDUs, or MSM). We did not consider women who inject drugs as most of the IDUs in India, except those in north-eastern states, are assumed to be men (Bill & Melinda Gates Foundation, 2009; Sarna et al., 2012; Solomon & Solomon, 2011). Moreover, women who have sex with women were not considered in the model because it is believed that this subgroup is almost nonexistent, at least in terms of its potential to drive the HIV epidemic in India (National AIDS Control Organization, 2006a). The MSM subgroup included homosexual (defined as men who only have sex with men/ transgendered people) and bisexual (defined as men who have sex with both men/transgendered people and women) men. It was necessary to make these distinctions in the MSM subgroup to account for the relatively higher HIV-risk behaviors observed among bisexual men as compared with their heterosexual counterparts in Mumbai (Hernandez et al., 2006).

It was assumed that individuals in the each subgroup interact with individuals in some (or all) of the other subgroups. These interactions defined the routes of transmission for each of the above-mentioned seven subgroups. For instance, HIV infection among FSWs was assumed to occur due to interaction (i.e., unprotected sex) with IDUs, heterosexual clients, and bisexual clients. Similarly, among MSM, HIV infection was assumed to occur as a result of unprotected sex with men/transgendered people or FSWs. The possible routes of transmission for each of the above subgroups considered in the model are presented in Table 1.

**Table 1:** Sub-groups and corresponding routes of transmission considered in the AEM

Subgroup	Routes of transmission
IDUs	IDUs
	Brothel-based FSWs
	Non-brothel based FSWs

FSWs (brothel and non-brothel-based FSWs)	IDUs
	Heterosexual clients
	Bisexual clients

Heterosexual clients	Brothel-based FSWs
	Non-brothel based FSWs

MSM (homosexual and bisexual men)	MSM
	Brothel-based FSWs
	Non-brothel-based FSWs

General women	Heterosexual regular male sexual partners
	Bisexual regular male sexual partner
	Casual male sexual partners (premarital, extramarital)

General men	Regular female sexual partners
	Casual female sexual partners (premarital, extramarital)

FSWs: Female sex workers; IDUs: Injecting drug users; MSM: men who have sex with men/ transgendered people

The key inputs used in the model included population size, sexual behaviors, injecting drug use and needle-sharing practices, prevalence of HIV and STDs, and ART coverage. Using these inputs, the model determined the HIV transmission probabilities (through unprotected vaginal sex, unprotected anal sex, and use of infected needle/ syringe) necessary to fit the observed epidemiological patterns, as seen in HIV sentinel surveillance data. Corrections in transmission probabilities were made in the presence of STDs or due to lack of male circumcision by adding cofactors that increased the effective transmission probability. The model then calculated the number of new infections in each of the subgroups through pre-defined routes of transmission. For example, the number of new HIV infections among clients infected by brothel-based FSWs during a year ’t’ was calculated as follows:

New HIV infections =[X_STD_ * F_STD_ (t)+ {1 - F_STD_ (t)}] * [Y_cc_ * F_cc_ (t)+ {1 - F_cc_ (t)}]* {(P_fm_ * S(t)) * (1 - C(t)} * (HIV_bbFSW_(t)) (1)

Where, X_STD_ represents the correction factor for STDs; F_STD_(t) is the STD prevalence among brothel-based FSWs in year ‘t’; Y_cc_ represents the correction factor for circumcision and F_cc_ is the fraction of men who are circumcised in year ‘t’; P_fm_ is the probability of HIV transmission from female to male per unprotected vaginal sex; S(t) is the average number of sexual contacts with clients for brothel-based FSWs in year ‘t’; C(t) is consistent condom use by brothel-based FSWs with clients in year ‘t’; and HIV_bbFSW_(t) denotes HIV prevalence among brothel-based FSWs in year ‘t’.

The quantities F_STD_(t), F_cc_, S(t), and C(t) were used as inputs and the transmission probabilities and adjustment factors for them (due to presence of STDs, lack of male circumcision) were calculated to get the best fit between the estimated and observed trend in HIV prevalence among various subgroups. The number of new HIV infections in each subgroup by different routes of transmission were similarly calculated. Specific outputs of the model included number of new, current and cumulative HIV infections and the mode of transmission for each of the subgroups specified in the model. A detailed description of the model and its application in other Asian countries is available elsewhere (Brown & Peerapatanapokin, 2004; Family Health International, 2008; Ma et al., 2012).

### 3.2 Inputs for the model

Inputs were provided for different time-points from 1980 to 2009. Values for the intermediate years for which data were not available were interpolated under assumption of linear change. The populations of men and women aged 15 and above over time were available from the decadal population census and from the district-level projections (Registrar General of India, 2001, 2011; United Nations Population Fund, 2009). Information on sexual behaviors and injecting drugs practices was taken from a series of cross-sectional surveys (Avert Society, 2004, 2009; Indian Council of Medical Research & Family Health International, 2007, 2011; National AIDS Control Organization, 2006b, 2006c). HIV prevalence among FSWs, MSM, IDUs, and ANC women was obtained from the HIV sentinel surveillance in Mumbai (India Health Action Trust, 2010). Based on global evidence that HIV prevalence among ANC women is an overestimate of HIV prevalence among general women (Brookmeyer, 2010; Gouws, Mishra, & Fowler, 2008), the observed HIV prevalence among ANC women was adjusted downward by a factor of 0.8 (Gouws et al., 2008). Key inputs used in the model along with the corresponding references are described in Table 2.

**Table 2:** Key inputs used in the Model

Indicators	Value (Year)	Reference
**Population size**		
Population aged 15 and above	5619685 (1981); 6997708 (1991); 8838672 (2001); 10720170 (2011); 11547837 (2016)	(Registrar General of India, 2001, 2011; United Nations Population Fund, 2009)
Sex- ratio (women per 1000 men) in the population aged 15 and above	708 (1981);777 (1991); 778 (2001); 789 (2011); 790 (2016)	(Registrar General of India, 2001, 2011; United Nations Population Fund, 2009)
Women in the age group 15-49 who are FSWs (%)	1.0 % (2001, 2008);	(Family Health International, 2001; India Health Action Trust, 2010)
FSWs who are brothel-based (%)	48% (2001); 32% (2008)	(Family Health International, 2001; India Health Action Trust, 2010)
Men in the age group 15-49 who are clients of FSWs (%)	2% (2006, 2008)	(India Health Action Trust, 2010; National AIDS Control Organization, 2006d)
Men in the age group 15-49 who are IDUs (%)	0.04% (2008)	(India Health Action Trust, 2010)
Men in the age-group 15-49 who are MSM (%)	2% (2008)	(National AIDS Control Organization, 2006d)

**Sexual behaviors of FSWs and their clients**		
Number of clients per week among brothel-based FSWs	18 (2001); 13 (2006, 2009);	(Family Health International, 2001; Indian Council of Medical Research & Family Health International, 2007, 2011)
Consistent condom use by brothel-based FSWs with clients (%)	71% (2004), 76% (2006), 95% (2009)	(Avert Society, 2004; Indian Council of Medical Research & Family Health International, 2007, 2011)
Average duration of working as an FSW	8 years (2006, 2009)	(Indian Council of Medical Research & Family Health International, 2007, 2011)
Number of clients per week among non-brothel based FSWs	18 (2001); 13 (2006, 2009);	(Family Health International, 2001; Indian Council of Medical Research & Family Health International, 2007, 2011)
Consistent condom use by non-brothel based FSWs with clients (%)	53% (2004), 64% (2006), 98% (2009)	(Avert Society, 2004; Indian Council of Medical Research & Family Health International, 2007, 2011)
Average duration for which men remain clients of FSWs	10 years (2006, 2009)	(Indian Council of Medical Research & Family Health International, 2007, 2011)

**Injecting drug practices**		
IDUs who share needles (%)	58% (2006); 53% (2009)	(Avert Society, 2009; National AIDS Control Organization, 2006c)
Number of injections used per day	2 (2001); 1 (2006)	(National AIDS Control Organization, 2006c)
Average duration of injecting drug use	15 years (2006)	(National AIDS Control Organization, 2006c)
IDUs who have sex with FSWs (%)	48% (2006, 2009)	(Avert Society, 2009; National AIDS Control Organization, 2006c)
Consistent condom use by IDUs with FSWs (%)	52% (2001); 58% (2006);	(National AIDS Control Organization, 2006c)
Consistent condom use by IDUs spouse or regular partners (%)	8% (2001); 13% (2006)	(National AIDS Control Organization, 2006c)

**Sexual behaviors with regular and casual sexual partners**		
Men who have sex with casual female partners (%)	9% (2006)	(National AIDS Control Organization, 2006d)
Women who have sex with casual male partners (%)	5% (2006)	(National AIDS Control Organization, 2006d)
Consistent condom use in casual sex (%)	53% (2006)	(National AIDS Control Organization, 2006d)
Consistent condom use in sex with spouse or regular partner (%)	27% (2006)	(National AIDS Control Organization, 2006d)

**Sexual behaviors of homosexual and bisexual men**		
MSM who have anal sex (%)	73% (2001); 80% (2006); 75% (2009)	(Avert Society, 2009; National AIDS Control Organization, 2006c)
Number of anal sex encounters in last week	2 (2001; 2006; 2009)	(Avert Society, 2009; National AIDS Control Organization, 2006c)
Average duration of same-sex behavior	15 years (2001, 2006; 2009)	(Avert Society, 2009; National AIDS Control Organization, 2006c)
Consistent condom use in anal sex with other MSM (%)	56% (2001), 79% (2006) & 80% (2009).	(Avert Society, 2009; National AIDS Control Organization, 2006c)
MSM who have sex with FSWs (%)	33% (2001), 26% (2006) & 33% (2009).	(Avert Society, 2009; National AIDS Control Organization, 2006c)
Consistent condom use by MSM with FSWs in past one year (%)	59% (2006) & 79% (2009).	(Indian Council of Medical Research & Family Health International, 2007, 2011)

**PLHA receiving ART**		
PLHA on ART (%)	27% (April, 2009)	(India Health Action Trust, 2010)

**HIV Prevalence (%)**		
FSWs	55.0% (2002), 54.3% (2003), 44.8% (2004), 30.5% (2005), 17.9% (2006), 19.4% (2007), 10.3% (2008)	(India Health Action Trust, 2010)
IDUs	39.4% (2002), 22.9% (2003), 29.2% (2004), 12.8% (2005), 20.4% (2006), 20.4% (2007), 20.0% (2008)	(India Health Action Trust, 2010)
MSM	16.8% (2002), 18.8% (2003), 9.6% (2004), 6.0% (2005), 7.6% (2006), 8.4% (2007), 9.2% (2008)	(India Health Action Trust, 2010)
ANC women	1.5% (2002), 1.3% (2003), 1.1% (2004), 1.2% (2005), 1.4% (2006), 1.0% (2007), 1.0% (2008)	(India Health Action Trust, 2010)

ANC: antenatal care; ART: Antiretroviral therapy FSWs: Female sex workers; IDU: Injecting drug users; MSM: Men who have sex with men/ transgendered people; PLHA: People living with HIV/ AIDS

## 3. Results

We estimated the probability of transmission for each unprotected vaginal sexual encounter from female to male as 0.12% and male to female as 0.18%; per unprotected anal sex encounter among MSM as 0.41%; and using infected needles/ syringes as 0.69%. The STD cofactor per sexual act for men in vaginal and anal sex was found to be 3.15 and 5.17 respectively, whereas this was estimated at 3.82 for females for vaginal sex. The increase in transmission probability due to lack of circumcision was estimated to be 1.6.

**Figure 1:**
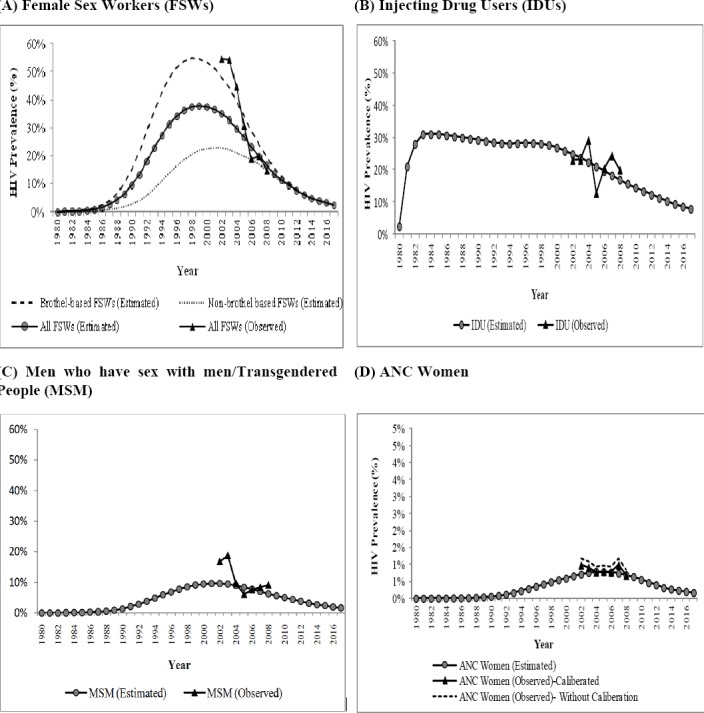
Estimated and observed HIV prevalence among different subgroups, Mumbai, 1980-2017

Figure 1 shows the estimated and observed trends in HIV prevalence among FSWs (total, brothel-based and non-brothel-based), IDUs, MSM, and ANC women. Differences between estimated and observed HIV prevalence among FSWs and MSM were high during the early 2000s, but narrowed from 2005 onwards. Prior to 2005, observed HIV prevalence among FSWs was closer to the estimated HIV prevalence among brothel-based FSWs. The estimated HIV prevalence among general women closely matched the calibrated HIV prevalence among ANC women. It may be noted that the scale for the figures for FSWs, MSM and IDUs was identical (ranging from 0% to 60%) whereas the scale for the figure for general women ranged from 0% to 5%. This was done to clearly represent estimated and observed HIV prevalence among general women who had very low HIV prevalence as compared to FSWs, MSM and IDUs.

Table 3 shows the trends in the number of new HIV infections among brothel-based FSWs, non-brothel based FSWs, heterosexual clients, IDUs, MSM, general women and general men from 2000-2017. Overall, the number of new HIV infections among adults was estimated to have reduced by 86% during 2000-2010. The greatest decline was estimated among FSWs and their heterosexual clients (95%-98%), followed by MSM (82%), general women (77%), IDUs (51%) and general men (42%). If HIV prevention efforts are continued with the same intensity, there would be about 4046 new HIV infections among adults during 2011-2017. Most of the new HIV infections would occur among general women (1666) and general men (977). Among the high-risk groups where the interventions are targeted, the maximum number of new HIV infections would occur among MSM (715) followed by heterosexual clients (213) and brothel-based FSWs (211).

**Table 3:** Estimated and projected number of new HIV infections among subgroups, Mumbai, 2000-2017

Year	Estimated Number of new HIV infections among							Total new HIV infections among adults

	Brothel-based FSWs	Non-brothel based FSWs	Heterosexual clients	IDUs	MSM	General women	General men	
2000	1003	586	2601	39	1004	2228	417	7878
2001	918	573	2313	36	983	2061	420	7304
2002	828	540	2017	34	882	1878	409	6588
2003	720	492	1692	33	758	1676	391	5762
2004	492	394	1128	29	585	1456	371	4455
2005	383	358	860	26	576	1229	346	3678
2006	289	371	631	24	519	1020	320	3174
2007	171	247	382	23	380	888	314	2405
2008	91	143	194	21	280	755	297	1781
2009	60	39	73	20	207	629	270	1298
2010	52	38	61	19	181	514	241	1106
2011	46	34	51	17	156	419	212	935
2012	40	30	42	16	135	338	184	785
2013	34	26	35	15	116	271	158	655
2014	29	23	28	14	98	217	135	544
2015	25	20	23	12	83	173	114	450
2016	20	17	19	12	70	138	95	371
2017	17	14	15	12	59	110	79	306

Reduction in number of new HIV infections (2000-2010) (%)			
	95%	94%	98%	51%	82%	77%	42%	86%

Number of new infections (2011-2017)								
	211	164	213	99	715	1666	977	4046

IDUs: Injecting drug users; FSWs: Female sex workers; MSM: Men who have sex with men/ transgendered people

Table 4 shows the trend in the percentage distribution of new HIV infections in each subgroup by routes of transmission at three points in time - 2000, 2010, and 2017. The proportion of new infections among IDUs and MSM from brothel-based FSWs reduced from nearly half (IDU: 44%; MSM: 54%) in 2000 to nearly one-fifth in 2010 (IDU: 16%; MSM: 20%). Brothel-based FSWs continue to be the source for most new infections among heterosexual clients (87% in 2000; 68% in 2010; 60% in 2017). The proportion of new infections among MSM due to unprotected sex with men and transgendered persons increased from 39% in 2000 to 61% in 2010;and is expected to increase to 75% in 2017. Although heterosexual clients were found to be a major source of infection among general women, bisexual men contributed to about 14% of new infections in this group in 2010; and this proportion is expected to increase to 19% in 2017.

**Table 4:** Percentage distribution of new HIV infections by routes of transmission among different subgroups at three time-points, Mumbai, 2000-2017

Subgroups and source of new HIV infections	Year		

	2000	2010	2017
New infections among IDUs from:			
IDUs (%)	51	73	83
Brothel-based FSWs (%)	44	16	8
Non-brothel based FSWs (%)	5	11	8

New infections among brothel-based FSWs from:			
IDUs (%)	1	6	12
Heterosexual clients (%)	93	63	47
Bisexual clients (%)	6	31	41

New infections among non-brothel-based FSWs from:			
IDUs (%)	1	8	14
Heterosexual clients (%)	94	42	29
Bisexual clients (%)	5	47	57

New infections among heterosexual clients of FSWs from:			
Brothel-based FSWs (%)	87	69	60
Non-brothel based FSWs (%)	12	25	27

New infections among MSM from:			
MSM (%)	39	63	75
Brothel-based FSWs (%)	54	20	14
Non-brothel-based FSWs (%)	6	17	11

New infections among general women from:			
Heterosexual regular male sexual partners (%)	79	75	67
Bisexual regular male sexual partners (%)	11	14	19
Casual male sexual partners (%)	8	10	11

New infections among general men from:			
Regular female sexual partners (%)	96	98	97
Casual female sexual partners (%)	4	2	3

FSWs: Female sex workers; IDUs: Injecting drug users; MSM: Men who have sex with men/ transgendered people

## 4. Discussion

This study shows that the number of new HIV infections among adults in Mumbai has reduced substantially during 2000-2010. The reduction has been most among FSWs and their heterosexual clients, followed by MSM, IDUs, general women and general men. Among the high-risk groups where HIV prevention programs in Mumbai are focused, MSM are projected to have the maximum number of new HIV infections during 2011-2017. The proportion of new HIV infections among FSWs and general women from bisexual men has increased during 2000-2010 and is projected to increase further during 2011-2017 if the current situation, in terms of program intensity and HIV risk behaviors of various subgroups, remains the same during this period.

The estimated transmission probabilities and cofactors for STDs and male circumcision are consistent with other studies (Baggaley, White, & Boily, 2010; Boily et al., 2009; Jin F et al., 2010; Kaplan & Heimer, 1992; Ward & Ronn, 2010). The estimated trend in HIV prevalence among FSWs and MSM was closer to the observed trends from 2005 onwards than in the previous years. It may be noted that the number of HIV sentinel surveillance sites among all subgroups dramatically increased across the country in 2005 and in subsequent years (National AIDS Control Organization, 2010), suggesting that observed HIV prevalence from 2005 onwards may be better representative of actual HIV prevalence in the various subgroups. This could be a possible reason for the better agreement in estimated and observed HIV prevalence in recent years. The estimated HIV prevalence among general women matched better with the calibrated values of observed HIV prevalence among ANC women than the observed values themselves. This was consistent with the fact that the HIV prevalence observed among ANC women tends to overestimate HIV prevalence among general women (Gouws et al., 2008). These results strengthen our hypothesis that the model adequately captured the key processes determining the transmission dynamics of HIV in Mumbai.

The estimated reduction in the number of new HIV infections in the adult population in Mumbai during 2000-2010 (86%) was higher than the reduction in the number of new HIV infections at the national level during 2000-2009 (56%) (National AIDS Control Organization, 2010). This could be explained, to some extent, by the following two reasons. First, the reference period for the two estimates is not exactly same. Second, the estimate of reduction in the number of new HIV infections at the national level represents an ’average reduction’, as it includes areas that experienced a greater reduction as well as those that reported a lesser reduction in the number of new HIV infections. As Mumbai received more intensive HIV prevention programs than many other parts of the country (Bill & Melinda Gates Foundation, 2008a; Chandrashekar et al., 2011; India Health Action Trust, 2010), and studies suggest greater success in HIV prevention with higher intensity programs than others (Moses et al., 2008), we argue that reduction in new HIV infections among adults in Mumbai may be higher than that observed at the national level, as seen in this study.

Findings suggest that HIV prevention programs were relatively less successful in preventing new HIV infections among MSM and IDUs than among FSWs and their heterosexual clients. In the case of MSM, this could be explained, at least partly, by the following factors. First, it has been found that as compared to working with FSWs, the HIV prevention program staff in Mumbai took longer to orient themselves on the nature of the MSM community and to build rapport with them (Chandrashekar et al., 2011). Also, the mean cost of delivering the intervention to MSM has been found to be higher than delivering it to FSWs in selected Indian cities, including Mumbai (Chandrashekar, et al., 2011). Additionally, MSM in India often face vulnerabilities such as lack of family acceptance, perceived need to be accepted by the general society, and a sexual identity crisis that are not adequately addressed by HIV prevention programs (Mysore Resettlement and Development Agency, 2010; Thomas et al., 2011). Unlike for other high-risk groups, there was no up-scaled HIV prevention program for IDUs in Mumbai, which could be a possible reason for less successful HIV prevention in this group (Basu, Joy, & Rathod, 2008; Bill & Melinda Gates Foundation, 2008a). Notably, the proportion of new infections among FSWs and general women from bisexual men is estimated to have increased over time. Although this increase is due to the faster decline in new HIV infections among FSWs and general women from heterosexual clients than the decline in the number of new HIV infections from bisexual men, it indicates that HIV prevention programs need to pay MSM special attention in Mumbai, which will also result in fewer HIV infections among FSWs and general women. It may be noted that we do not intend to suggest a relaxation in HIV prevention program efforts among other high-risk groups, especially FSWs and their heterosexual clients, which have shown great success in the recent past.

Notably, the results of this study indicate a substantial number of new HIV infections among general women and general men despite considerable program success in reducing new HIV infections among high-risk groups. New HIV infections among general women are estimated to occur mainly from heterosexual clients and bisexual men whereas new HIV infections among general men are estimated to occur mainly from casual sexual relationships that are non-commercial in nature. This suggests the need for early detection of HIV infection among men and women in the general population and for ensuring consistent condom use by those who are HIV-positive. With the increasing number of HIV testing and counseling facilities in Mumbai (India Health Action Trust, 2010), it may be possible to prevent many of these infections among general women and men. Efforts to strengthen the network of people living with HIV may also help in mobilizing and reaching HIV-positive men and women and hence to motivate them for greater compliance for consistent condom use.

## 5. Conclusions

In conclusion, this study shows that HIV prevention programs have reduced the number of new HIV infections substantially among adults in Mumbai. The reduction in the number of new HIV infections has been more among FSWs and their heterosexual clients than that among MSM and IDUs. The tremendous success of the program in preventing new HIV infections among FSWs and their heterosexual clients has resulted in an epidemiological shift in the HIV epidemic in Mumbai. The ability of the HIV prevention program in Mumbai to prevent most of the new HIV infections among FSWs and their heterosexual clients has shifted the focus of the epidemic from FSWs and their heterosexual clients to MSM and the general population. In order to further reduce new HIV infections, the HIV prevention program for FSWs and their heterosexual clients should be continued with the same intensity, and the strategies to prevent new infections among MSM and the general population should be revised and strengthened. HIV prevention efforts among MSM will not only reduce new HIV infections in the MSM community, but also among brothel-based FSWs and general women. Ensuring early detection of HIV, and increasing the level of consistent condom use by HIV-infected men and women are essential to prevent most of the new HIV infections among adults in Mumbai.

## References

[ref1] (2004). Avert Society. Behavioral Surveillance Survey in Maharashtra: Wave II.

[ref2] (2009). Avert Society. Behavioral Surveillance Survey: Maharashtra.

[ref3] Baggaley R. F, White R. G, Boily M. C (2010). HIV transmission risk through anal intercourse: systematic review, meta-analysis and implications for HIV prevention. Int J Epidemiol.

[ref4] Basu J. K, Joy A, Rathod N. J (2008). Needle sharing and risky sexual behavioral among IDUs in a high prevalence metropolitan city of Mumbai in Western India: need to upscale focused interventions now. Paper presented at the AIDS 2008 - XVII International AIDS Conference, Mexico. Abstract no. MOPE0592.

[ref5] (2008a). Bill & Melinda Gates Foundation. Avahan, the India AIDS Initiative - the Business of HIV prevention at Scale.

[ref6] (2008b). Bill & Melinda Gates Foundation. Off the Beaten Track: Avahan’s Experience in the Business of HIV Prevention among India’s Long-Distance Truckers.

[ref7] (2009). Bill & Melinda Gates Foundation. From Hills to Valleys: Avahan’s HIV Prevention Program among Injecting Drug Users in Northeast India.

[ref8] Boily M. C, Baggaley R. F, Wang L, Masse B, White R. G, Hayes R. J, Alary M (2009). Heterosexual risk of HIV-1 infection per sexual act: systematic review and meta-analysis of observational studies. Lancet Infect Dis.

[ref9] Brookmeyer R (2010). Measuring the HIV/AIDS epidemic: approaches and challenges. Epidemiol Rev.

[ref10] Brown T, Peerapatanapokin W (2004). The Asian Epidemic Model: a process model for exploring HIV policy and programme alternatives in Asia. Sex Transm Infect.

[ref11] Chandrashekar S, Vassall A, Reddy B, Shetty G, Vickerman P, Alary M (2011). The costs of HIV prevention for different target populations in Mumbai, Thane and Bangalore. BMC Public Health.

[ref12] Churi C, Anjenaya S (2010). Sexual behaviour among truck drivers halting at Kalamboli Truck Terminal, Navi Mumbai. Australasian Medical Journal.

[ref13] (2001). Family Health International. Mapping of commercial sex access points and relevant service outlets in Maharashtra, 2001. New Delhi.

[ref14] (2008). Family Health International. The Asian Epidemic Model (AEM) Projections for HIV/AIDS in Thailand: 2005-2025.

[ref15] Gouws E, Mishra V, Fowler T. B (2008). Comparison of adult HIV prevalence from national population-based surveys and antenatal clinic surveillance in countries with generalised epidemics: implications for calibrating surveillance data. Sex Transm Infect.

[ref16] Hernandez A. L, Lindan C. P, Mathur M, Ekstrand M, Madhivanan P, Stein E. S, Jerajani H. R (2006). Sexual behavior among men who have sex with women, men, and Hijras in Mumbai, India--multiple sexual risks. AIDS Behav.

[ref17] (2010). India Health Action Trust. HIV/AIDS Situation and Response in Maharashtra: Epidemiological Appraisal Using Data Triangulation.

[ref18] (2007). Indian Council of Medical Research, & Family Health International. National Interim Summary Report-India, Integrated Behavioral and Biological Assessment (IBBA), Round 1.

[ref19] (2011). Indian Council of Medical Research, & Family Health International. National Interim Summary Report-India, Integrated Behavioral and Biological Assessment (IBBA), Round 2 (2009-10).

[ref20] Jain M. K, John T. J, Keusch G. T (1994). A review of human immunodeficiency virus infection in India. J Acquir Immune Defic Syndr.

[ref21] Jin F, Jansson J, Law M, Prestage G. P, Zablotska I, Imrie J. C, Wilson D. P (2010). Per-contact probability of HIV transmission in homosexual men in Sydney in the era of HAART. AIDS.

[ref22] Kaplan E. H, Heimer R (1992). A model-based estimate of HIV infectivity via needle sharing. J Acquir Immune Defic Syndr.

[ref23] Ma N, Zheng M, Liu M, Chen X, Zheng J, Chen H.-g, Wang N (2012). Impact of Condom Use and Standardized Sexually Transmitted Disease Treatment on HIV Prevention Among Men Who Have Sex with Men in Hunan Province: Using the Asian Epidemic Model. AIDS Res Hum Retroviruses.

[ref24] Moses S, Ramesh B. M, Nagelkerke N. J, Khera A, Isac S, Bhattacharjee P, Blanchard J. F (2008). Impact of an intensive HIV prevention programme for female sex workers on HIV prevalence among antenatal clinic attenders in Karnataka state, south India: an ecological analysis. AIDS.

[ref25] (2010). Mysore Resettlement and Development Agency. Targeted HIV Interventions for MSM Communities – are the current strategies working?. Rural Management Systems Series, Paper – 56.

[ref26] (2006a). National AIDS Control Organization. NACP-III - To halt and reverse the HIV epidemic in India.

[ref27] (2006b). National AIDS Control Organization. National Behavioral Surveillance Survey, 2006, Female sex workers and clients of female sex workers.

[ref28] (2006c). National AIDS Control Organization. National Behavioral Surveillance Survey, 2006, Men who have Sex with Men (MSM) and Injecting Drug Users (IDUs).

[ref29] (2006d). National AIDS Control Organization. National Behavioral Surveillance Survey: General Population.

[ref30] (2010). National AIDS Control Organization. Annual Report, 2009-10.

[ref31] (2001). Registrar General of India. 2001 Census Data.

[ref32] (2011). Registrar General of India. Provisional Population Totals- Maharashtra- Data sheet.

[ref33] Saggurti N, Schensul S. L, Verma R. K (2009). Migration, mobility and sexual risk behavior in Mumbai, India: mobile men with non-residential wife show increased risk. AIDS Behav.

[ref34] Saggurti N, Verma R. K, Jain A, Achyut P, Ramarao S (2008). Patterns and Implications of Male Migration for HIV Prevention Strategies in Maharashtra, India. Technical Brief from Population Council India: Number 3, Population Council.

[ref35] Sarna A, Tun W, Bhattacharya A, Lewis D, Singh Y. S, Apicella L (2012). Assessment of unsafe injection practices and sexual behaviors among male injecting drug users in two urban cities of India using respondent driven sampling. The Southeast Asian Journal of Tropical Medicine And Public Health.

[ref36] Schensul S. L, Mekki-Berrada A, Nastasi B. K, Singh R, Burleson J. A, Bojko M (2006). Men’s extramarital sex, marital relationships and sexual risk in urban poor communities in India. J Urban Health.

[ref37] Sgaier S. K, Claeson M, Gilks C, Ramesh B. M, Ghys P. D, Wadhwani A, … K, C (2012). Knowing your HIV/AIDS epidemic and tailoring an effective response: how did India do it?. Sex Transm Infect.

[ref38] Singh D. P (2006). Slum Population In Mumbai: Part I. Published in IIPS ENVIS Center.

[ref39] Solomon S. S, Solomon S (2011). HIV serodiscordant relationships in India: translating science to practice. Indian J Med Res.

[ref40] (2012). The Humsafar Trust. http://www.humsafar.org/.

[ref41] Thomas B, Mimiaga M. J, Kumar S, Swaminathan S, Safren S. A, Mayer K. H (2011). HIV in Indian MSM: reasons for a concentrated epidemic & strategies for prevention. Indian J Med Res.

[ref42] (2009). United Nations Population Fund. District Level Population Projections in Selected States of India – 2006 to 2016.

[ref43] Verma R. K, Saggurti N, Singh A. K, Swain S. N (2010). Alcohol and sexual risk behavior among migrant female sex workers and male workers in districts with high in-migration from four high HIV prevalence states in India. AIDS Behav.

[ref44] Ward H, Ronn M (2010). Contribution of sexually transmitted infections to the sexual transmission of HIV. Curr Opin HIV AIDS.

[ref45] Zahiruddina Q. S, Gaidhanea A. M, Shanbhagb S, Zodpeyb S. P (2011). High-risk sexual partnerships and condom use among truckers entering Mumbai city. Int J Biol Med Res.

